# Enlargement of the Prostate.—IV

**Published:** 1909-06-12

**Authors:** 


### SURGERY.

### ENLARGEMENT OF THE PROSTATE.—IV.

The ultimate success of the operation of prostatectomy depends largely upon the after treatment of the case, or, in other words, upon the constant care which must be exercised in keeping the dressings fresh and in seeing that the kidneys are encouraged to do their work properly. There is no doubt that the operation is apt to throw a sudden strain on the kidneys, and it not infrequently happens that, although they were excreting a sufficient amount of urea previously, they do not adequately perform their function afterwards. To avert this it is well to apply a linseed poultice to each loin immediately after the operation, and to keep one on for, say, the first twenty-four hours. This treatment is simple but effective. The patient should be kept flat on his back until he comes round from the anæsthetic; but as soon as he has recovered he should be propped up a little, because, in view of his advanced age, hypostatic pneumonia is quite likely to ensue if this precaution is not taken.

The main trouble which has to be combated is the constant wetting of the dressings with the urine, which makes its way out of the wound. To overcome this several ingenious mechanical appliances have been invented. The principle, which is similar in all of them, is much the same as that of a colotomy apparatus, a celluloid cup being fixed over the wound to collect the urine, which is led away through small indiarubber tubes which are attached to openings in its lower part. The disadvantage of these appliances is that it is very difficult to get them to work properly, and if there is any leakage they are worse than useless.

The writer's experience is that ordinary dressings are quite satisfactory if a thick pad of wood-wool is applied outside the gauze. It will be found that it is unnecessary to change this more than three or four times in the twenty-four hours. The skin round the wound should be covered for a considerable distance with a thickish layer of ointment to prevent excoriation owing to its being constantly wet with urine. The best ointment for this purpose is a mixture of equal parts of lanoline and unguentum zinci. But the secret of success is to apply enough of it, and to spread it over a sufficiently wide area.

The wound should be dressed by the surgeon at least once daily, and the empyema tube gradually shortened. On the third day it should be replaced by one of a smaller size, and on the fourth or fifth day this may be dispensed with altogether. By this time a track will have been made down to the bladder, which remains open of its own accord.

Some surgeons leave a catheter in the urethra at the end of the operation, in order that they may irrigate the bladder through it during the subsequent dressings. Opinions differ upon the advisability of doing this. The writer is opposed to it as a routine practice, and for the following reasons: There does not appear to be any advantage in irrigating a bladder per urethram when it is already well drained by a suprapubic opening, and to do so causes the patient some little discomfort daily. Besides, after a few days a mucopurulent discharge collects between the catheter and the urethral wall, and it stands to reason that its presence must militate against the healing of the cavity from which the prostate has been removed.

On the other hand, experience shows that the less the patient is disturbed by such manipulations, the more complete and rapid is his recovery. In fact, it is really not necessary to pass a urethral instrument at all during the whole period of convalescence. In a successful case the suprapubic wound begins to contract down as soon as the tube is removed, until at about the end of a fortnight it is almost completely closed. As soon as the resistance to the passage of urine through the suprapubic sinus is greater than it is per urethram the patient will pass water naturally. This usually occurs between the fourteenth and eighteenth days after the operation.

Certain complications must be mentioned which interfere with a successful result, and if urine is not passed naturally in three weeks' time it is probable that one of them is responsible. Thus phosphatic calculi have been known to form in the cavity from which the prostate has been removed. Their nucleus is formed by small tags of blood-clot which are attached to the well of the cavity, and calculi are formed by the successive deposits upon them of layers of the phosphate which separates out from the urine.

It is well known that phosphates quickly settle on a foreign body in the bladder, and in this case the clotted blood acts in this capacity. The passage of a catheter will disclose the state of affairs, because the calculi will be felt as the point of the catheter passes into the bladder. If calculi are present there is no alternative but to open up the suprapubic wound and remove them. In this case the bladder should be subsequently irrigated daily in order to prevent a reaccumulation. Sometimes the natural flow of urine is prevented by a small tag which has been left on the wall of the prostatic cavity, and this may fall down and occlude the orifice of the urethra, just as a "middle-lobe enlargement" does.

It is always exceedingly hard to diagnose this condition without having recourse to cystoscopy. Cases have also been recorded in which obstruction to the outflow of urine occurs some time after the operation owing to the contraction of the prostatic cavity, so that a fibrous stricture results. The writer has not seen such a case, and in view of the width of the cavity one would think that such a complication would be very rare.

The best treatment for this state of affairs consists in dilatation, as in the case of an ordinary stricture. Occasionally after the operation the patient loses urinary control altogether. Whether this is due to the disturbance with the normal anatomy of the parts at the neck of the bladder or to damage to the compressor urethræ at the operation is uncertain. But, whatever the cause, the prognosis with regard to recovery of control is exceedingly bad.

